# Exploring Adiponectin in Autosomal Dominant Kidney Disease: Insight and Implications

**DOI:** 10.3390/genes15040484

**Published:** 2024-04-11

**Authors:** Ersilia Nigro, Marta Mallardo, Maria Amicone, Daniela D’Arco, Eleonora Riccio, Maurizio Marra, Fabrizio Pasanisi, Antonio Pisani, Aurora Daniele

**Affiliations:** 1 CEINGE-Biotecnologie Avanzate Scarl “Franco Salvatore”, Via G. Salvatore 486, 80145 Naples, Italy; ersilia.nigro@unicampania.it (E.N.); darco@ceinge.unina.it (D.D.); 2 Dipartimento di Scienze e Tecnologie Ambientali, Biologiche, Farmaceutiche, Università della Campania “Luigi Vanvitelli”, Via Vivaldi 43, 81100 Caserta, Italy; 3 Dipartimento di Medicina Molecolare e Biotecnologie Mediche, Università degli Studi “Federico II”, Via Pansini 5, 80131 Naples, Italy; marta.mallardo@unicampania.it; 4 Unità di Nefrologia, Dipartimento di Sanità Pubblica, Università di Napoli “Federico II”, Via Pansini 5, 80131 Naples, Italy; maria.amicone@unina.it (M.A.); eleonora.riccio@unina.it (E.R.); antonio.pisani@unina.it (A.P.); 5 Department of Clinical Medicine and Surgery, University of Naples “Federico II”, Via Pansini 5, 80131 Naples, Italy; maurizio.marra@unina.it (M.M.); fabrizio.pasanisi@unina.it (F.P.)

**Keywords:** adiponectin, ADPKD1-2, SNPs, ADIPOQ, PPARγ

## Abstract

Autosomal Dominant Polycystic Kidney Disease (ADPKD) is a common monogenic disorder characterized by renal cysts and progressive renal failure. In kidney diseases, adipose tissue undergoes functional changes that have been associated with increased inflammation and insulin resistance mediated by release of adipokines. Adiponectin is involved in various cellular processes, such as energy and inflammatory and oxidative processes. However, it remains to be determined whether adiponectin is involved in the concomitant metabolic dysfunctions present in PKD. In this scenario, we aimed to analyze: (a) PPARγ, ADIPOQ, ADIPOR1 and ADIPOR2 gene variations in 92 ADPKD patients through PCR-Sanger sequencing; and (b) adiponectin levels and its oligomerization state by ELISA and Western Blot. Our results indicated that: (a) 14 patients carried the PPARγ SNP, 29 patients carried the ADIPOQ SNP rs1501299, and 25 patients carried the analyzed ADIPOR1 SNPs. Finally, 82 patients carried ADIPOR2 SNPs; and (b) Adiponectin is statistically lower in ADPKD patients compared to controls, and further statistically lower in ESRD than in non-ESRD patients. An inverse relationship between adiponectin and albumin and between adiponectin and creatinine and a direct relationship between adiponectin and eGFR were found. Interestingly, significantly lower levels of adiponectin were found in patients bearing the ADIPOQ rs1501299 SNP and associated with low levels of eGFR. In conclusion, adiponectin levels and the presence of ADIPOQ rs1501299 genotype are significantly associated with a worse ADPKD phenotype, indicating that both could potentially provide important insights into the disease. Further studies are warranted to understand the pathophysiological role of adiponectin in ADPKD patients.

## 1. Introduction

Autosomal Dominant Polycystic Kidney Disease (ADPKD) is a common monogenic disorder with an increasing prevalence in the last decade [1,2]. The patients affected by ADPKD are characterized by progressive formation of cysts in the kidneys with progressive renal failure; in addition, more than half suffer from an increased predisposition to atherosclerosis, chronic inflammation, and show a higher risk of cardiovascular disease [3]. Although a fully reliable disease marker is still missing, several biochemical markers that can be assayed in patients’ blood and urine such as albumin, creatinine and total proteins [4]. In the majority of patients, the cause of disease is a mutation in the PKD1 (±85%) or PKD2 (±15%) genes that encode the polycystin-1 or polycystin-2 proteins, respectively [5,6].

The molecular mechanisms involved are not yet well understood, but obesity represents a condition that contribute to the risk of developing chronic renal failure, even in the absence of any other metabolic dysregulation [7]. The metabolic changes that adipose tissue undergoes in kidney disease are associated with increased inflammation and insulin resistance mediated by adipokine release, activation of the renin-angiotensin aldosterone system (RAAS), and oxidative stress [8].

Among adipokines, adiponectin is a cytokine of 30 kDa, accounting for approximately 0.01% of total serum proteins [9]. Adiponectin assembles to form three major oligomeric forms: the trimers (LMW), the hexamers (MMW), and the high-molecular-weight (HMW) [10]. Initially studied for its metabolic functions (insulin sensitization and enhancement of fatty acid oxidation), adiponectin has attracted attention for additional functions such as the inhibition of inflammatory and oxidative processes [11]. Consequently, serum adiponectin levels have been found modulated in both metabolic (obesity and related disorders) and immune and inflammatory disorders [12,13,14].

In obesity, reduced adiponectin levels have been associated with insulin resistance, cardiovascular diseases and obesity related kidney diseases. On the other hand, patients with chronic kidney disease have dysregulated levels of adiponectin which correlate with disease progression [15,16,17,18]. More importantly, the adiponectin involvement in renal alterations has been demonstrated independently of the presence of obesity, suggesting a role for this adipokine in kidney patho-physiology [19]. Furthermore, adiponectin is inversely related to renal function, and represents a predictor of end-stage renal disease with a strong association with disease severity and mortality [20]. Moreover, serum adiponectin levels are elevated in patients with albuminuria and are positively correlated with urine albumin/creatinine ratio [18].

Adiponectin is encoded by the ADIPOQ gene located on chromosome 3q27 [21]. Genetic variations in the ADIPOQ gene have been found associated with adiponectin expression in several studies and in various pathologies [22,23,24]. Currently, only one study has examined the distribution of the ADIPOQ rs1501299, which significantly differs between CKD and controls [25]. Adiponectin exerts its biological effects mainly by ADIPOR1 and ADIPOR2 receptors, which are expressed in the kidneys and whose activation could prevent or improve diabetic nephropathy [26]. Furthermore, both ADIPORs have been linked with a high risk of CVD. In addition, Peroxisome proliferator-activated receptor gamma (PPARγ), which regulates transcription of ADIPOQ gene, is associated with increased adiponectin levels and a reduced risk of type 2 diabetes [27].

In this scenario, our study was aimed to investigate the ADIPOQ, ADIPOR1 and ADIPOR2, PPARγ genetic variations in 92 ADPKD patients. In addition, the adiponectin serum levels were tested and compared to a sex-, BMI- and age-matched group of 104 healthy controls. Finally, the oligomerization state of adiponectin was investigated. 

To our knowledge, while the amount literature about the role of adiponectin in several renal disorders is increasing, no data are available about the regulation of adiponectin levels in PKD patients. Finally, the most relevant SNPs in the genes encoding for ADIPOQ, Adiponectin receptors 1, 2 (ADIPOR1, ADIPOR2) and PPARγ were investigated.

## 2. Materials and Methods

### 2.1. Patients Selection 

This is a secondary analysis of the previous published paper [28]; here, 92 patients (mean age 42.84 ± 14.7) diagnosed with ADPKD were recruited from Nephrology Unit, Department of Public Health, “Federico II” University, Naples, and Hospital outpatient clinic, “Federico II”, Naples. The study inclusion criteria and the genetic characterization of ADPKD patients has been previously performed [28]. Briefly, the inclusion criteria were either with a positive family history of PKD and met the unified criteria for ultrasonographic diagnosis of PKD; or were without PKD family history, but had confirmed renal cysts through imaging as per the unified criteria for ultrasonographic diagnosis of PKD [28]. Supplementary Table S1 shows the pathogenic variants found in the selected population. Blood samples were collected after a 12 h overnight fasting period and centrifuged to collect serum. Serum aliquots were immediately frozen in liquid nitrogen and stored at −80 °C. The following clinical and biochemical values were recorded (see Table 1): BMI, total cholesterol, triglycerides, glycemia, AST, ALT, WBC, neutrophils %, lymphocytes%, NLR, RBC, HGB, EGFR.

A total of 104 healthy volunteers, aged 45.91 ± 16.6 years, constituted the control group. The research protocol was conducted in accordance with the principles of the Helsinki II Declaration; according to the current legislation in Italy, informed consent was obtained from each subject. The protocol of this study was approved by the Federico II Ethical Committee (protocol code 2018-000477-77, date of approval: 16 November 2022).

### 2.2. ELISA

Total serum adiponectin concentration was measured by enzyme-linked immunosorbent assay (ELISA) utilizing a polyclonal antibody, in house produced, versus a human adiponectin sequence region (H2N-ETTTQGPGVLLPLPKG-COOH), as previously reported [29]. Each sample was tested three times in duplicate.

### 2.3. Western Blotting 

Serum samples were quantified for total proteins by Bradford’s method (Bio-Rad, Hercules, CA, USA); 5 µg of total proteins were treated with 1× Laemmli buffer, heated at 95 °C for 5 min and loaded under non-reducing conditions on 10%SDS-PAGE gel and transferred as previously described [30]. The blots were scanned by using ChemiDoc MP imaging system (Bio-Rad, Hercules, CA, USA) and analyzed by densitometry with ImageJ software (version 1.53) (http://rsbweb.nih.gov.ij/), (accessed on 1 October 2023). A representative sample of 30 patients and 30 healthy controls was analyzed and tested two times in duplicate.

### 2.4. SNPs Analysis by Sanger Sequencing 

Genomic DNA was amplified by singleplex PCR using the FailSafe PCR System (Epicentre, Madison, WI, USA). The following SNPs have been analyzed in the entire population of ADPKD patients through Sanger sequencing: ADIPOQ c.45T>C; ADIPOQ c.268G>A; ADIPOQ c.214+62G>T PPARγ c.34C>G; AdipoR1 c.94-12A>G; AdipoR1 c. 94-8T>G; AdipoR2 c.650+20G>A; AdipoR2 c.*1642C>T; AdipoR2 c.*1718C>T; AdipoR2 c.795G>A; AdipoR2 171+48A>T; (Supplementary Table S2). Universally tagged sequencing primers were designed using the following software: Primer3 version 1.1.4 (http://www.sourceforge.net; accessed on 1 October 2023). Primers are available on request. Thermal cycling was performed with 15 cycles [30 s at 98 °C; 30 s at 62 °C (−0.5 °C each cycle); 60 s at 72 °C], followed by 15 cycles (30 s at 98 °C; 30 s at 55 °C; 60 s at 72 °C).

### 2.5. Statistical Analysis 

The data were analyzed with SPSS software. (Version 27.0, IBM Corp, Armonk, NY, USA). Results are presented as mean ± standard deviation (SD), and *p* < 0.05 was considered statistically significant. To compare groups (ADPKD vs. Control), we used unpaired *t*-tests. Furthermore, the adiponectin levels were adjusted for weight, age, BMI, systolic and diastolic pressure, and then compared between groups (ADPKD and Controls). Pearson correlation (r) was used to evaluate the linear correlation between different variables.

## 3. Results

### 3.1. Biochemical and Clinical Parameters of ADPKD Patients Compared to Controls

The baseline characteristics of the study participants are shown in Table 1. There were no statistically significant differences in age, sex, BMI, diastolic pressure, glycemia, total cholesterol, triglycerides between the two groups. Neutrophils, NLR and systolic pressure values were statistically higher in ADPKD patients compared to controls (*p* value < 0.001), indicating an inflammatory state in patients. Albumin and lymphocytes values were lower in patients than in controls (*p* value = 0.01). As expected, creatinine, uric acid, and urea were higher in patients compared to controls (*p* value < 0.001).

### 3.2. Adiponectin Evaluation and Oligomerization State

**Figure 1:**
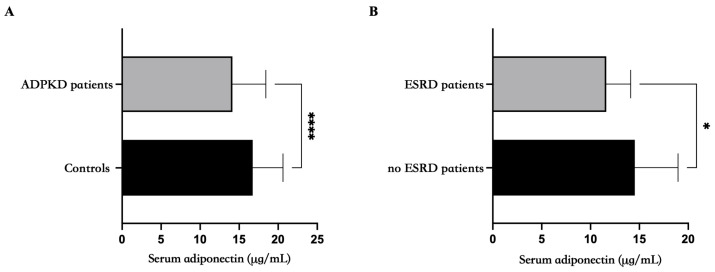
Total serum adiponectin is decreased in ADPKD patients compared to controls. (**A**) Adiponectin levels were statistically reduced in patients with ADPKD compared to controls (14.13 ± 4.2 vs. 16.73 ± 3.8 µg/mL; *p* value < 0.0001) (**B**) Adiponectin levels were statistically lower in a subgroup of ADPKD patients at ESRD vs. age-matched ADPKD patients not in ESRD (13.74 ± 4.40 vs. 15.65 ± 3.58 µg/mL; *p* value = 0.02). * *p* < 0.05. **** *p* < 0.0001.

Adiponectin levels were statistically lower in ADPKD patients compared to healthy controls (14.13 ± 4.2 vs. 16.73 ± 3.8 µg/mL; *p* value < 0.0001) (Figure 1A). Furthermore, adiponectin levels were adjusted for weight, age, BMI, systolic and diastolic pressure and then compared between the groups (ADPKD and controls); adiponectin levels were still lower in ADPKD patients than in controls (Table 2).

Interestingly, adiponectin was further statistically lower in ESRD than in non-ESRD patients (11.62 ± 2.5 vs. 14.54 ± 4.4 µg/mL; *p* value = 0.04) (Figure 1B). 

**Figure 2:**
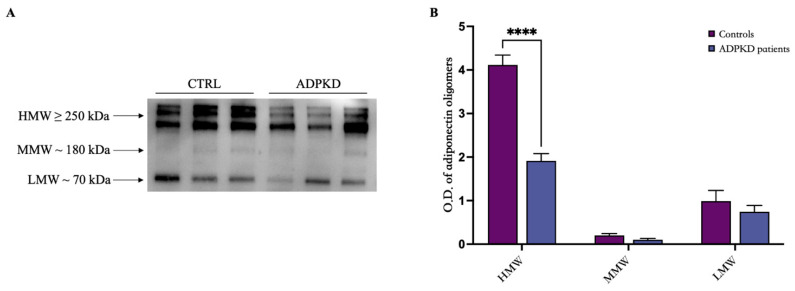
Oligomeric distribution of adiponectin in ADPKD patients and healthy controls. (**A**) Representative Western blot image for adiponectin oligomers, HMW, MMW, and LMW in the serum of controls and patients diagnosed with ADPKD. (**B**) Graphical representation of pixel quantization of analyzed controls and ADPKD patients. **** *p* < 0.0001.

Additionally, we analyzed the oligomeric distribution of adiponectin in ADPKD patients compared to healthy controls by Western blotting (Figure 2). Three bands corresponding to HMW (≥250 kDa), MMW (~180 kDa), and LMW (~70 kDa) oligomers were evident for both controls and ADPKD patients (Figure 2A). As shown in Figure 2B, the densitometric analysis of oligomeric distribution showed that ADPKD patients had a lower expression of HMW oligomers compared to controls.

**Figure 3:**
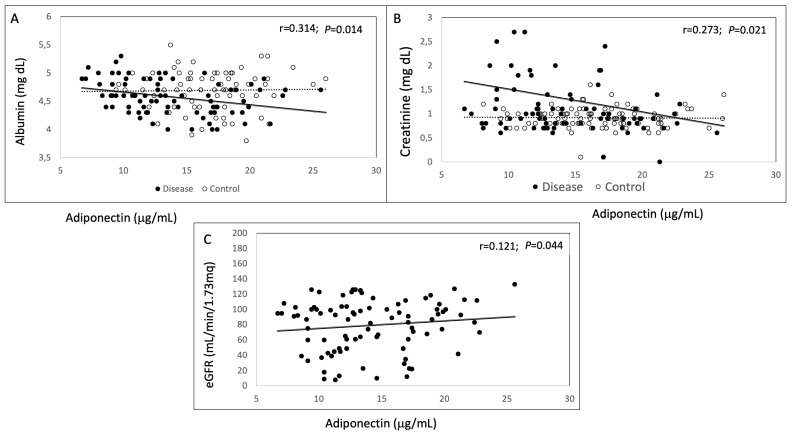
Correlation analysis between adiponectin and clinical parameters in ADPKD patients and controls. Dashed lines indicate control subjects while dark lines refer to patients. Adiponectin inversely correlated with albumin (**A**), and creatinine (**B**) in both ADPKD patients and controls. Adiponectin positively correlated with eGFR (**C**) in ADPKD patients.

Subsequently, within patients, we analyzed adiponectin correlations with several clinical parameters to evaluate its relationship with disease prognosis. We found an inverse relationship between adiponectin and albumin (*p* = 0.014) (Figure 3A) and an inverse relationship between adiponectin and creatinine (*p* = 0.021) (Figure 3B). A direct relationship between adiponectin and eGFR was found (*p* = 0.04) (Figure 3C). Altogether, these correlations suggest that adiponectin is inversely related to disease severity and poor prognosis.

Successively, we divided patients into three groups, defining three classes of severity, according to the presence of the following parameters: BMI > 25, creatinine > 1.1 mg/dL, systolic pressure > 120, diastolic pressure > 80, urea > 43 mg/dL, uric acid > 6 mg/dL, NLR > 3, eGFR < 90. According to each parameter, the three groups were divided as follows: 1 mild (0–1 point), 2 moderate (2–4 points), 3 severe (>4 points). Adiponectin distribution within the three groups supported the evidence that it correlates with disease seriousness: indeed, group 1 had higher levels of adiponectin (14.88 ± 5.1) compared to group 2 (13.60 ± 3.2) and group 3 (13.94 ± 4.2).

### 3.3. SNPs Analysis in PPARγ, ADIPOQ, ADIPOR1 and ADIPOR2 Genes

The SNP analysis, performed by PCR and Sanger sequencing, revealed 14 patients bearing the PPARγ rs1801282 SNP (Table 3). Regarding ADIPOQ, we found in 26, 4 and 29 patients with the following SNPs: rs2241766, rs62625753, rs1501299. Further, 23 and 25 patients bore the analyzed ADIPOR1 SNPs, rs2275737 and rs2275738, respectively. Finally, 18, 23, and 16 patients carried the following ADIPOR2 SNPs: rs16928751, rs1044471, rs12342 (see Table 3).

**Figure 4:**
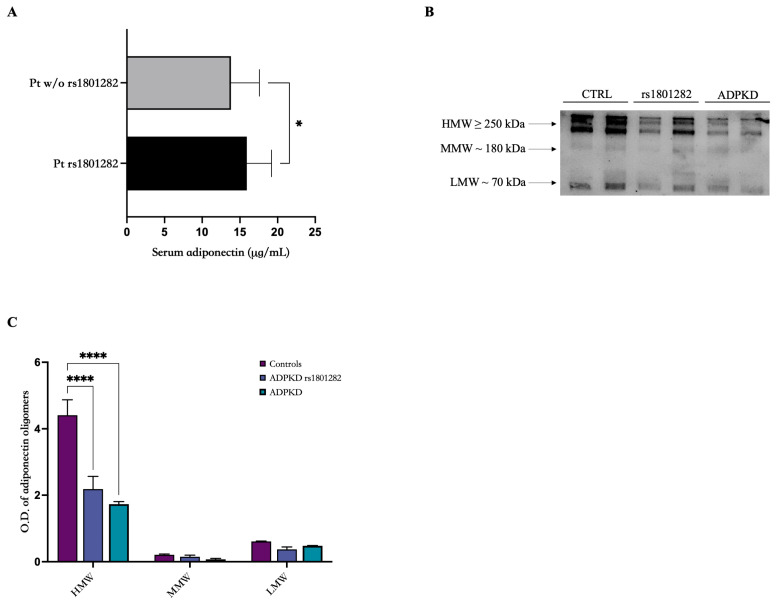
Serum total adiponectin is expressed differently among ADPKD patients with SNPs in PPARγ and ADIPOQ genes. (**A**) Patients bearing the PPARγ rs1801282 SNP had higher adiponectin levels compared to the non-mutated ones (15.92 ± 3.27 vs. 13.81 ± 3.76 µg/mL; *p* value = 0.04). (**B**) The oligomeric state of adiponectin was also analyzed. (**C**) Patients bearing the PPARγ rs1801282 SNP showed an oligomeric profile comparable to that of control subjects and non-mutated patients. * *p* < 0.05. **** *p* < 0.0001.

When we analyzed adiponectin levels and the main clinical parameters in those patients with SNPs, we found that patients bearing the PPARγ rs1801282 SNP had significantly higher adiponectin levels than the non-mutated patients (15.92 ± 3.27 vs. 13.81 ± 3.76 µg/mL; *p* value = 0.04) (Figure 4A). Interestingly, when we considered ADIPOQ mutated patients, no significant modulation of adiponectin levels was found in the SNPs patients groups.

Subsequently, we analyzed the oligomeric profile of those patients bearing the PPARγ rs1801282 SNP with healthy controls (Figure 4B,C). As shown in panel B, the oligomeric profile was comparable among the groups, with evidence for a modulation of adiponectin total levels in the three groups (both PPARγ and ADIPOQ had lower levels than controls while PPARγ higher than ADIPOQ). 

## 4. Discussion

In this study, conducted comparing an ADPKD Italian cohort to a healthy volunteer group, we analyzed adiponectin levels and its oligomerization state in relation to biochemical, genetic, and clinical parameters.

To date, numerous studies have been published providing details on the association between adiponectin and kidney function [15,16,17,18,31]. In detail, serum adiponectin concentrations are higher in patients with renal insufficiency [32]. Much of the previous work on the association between adiponectin and CKD has produced conflicting results, and has originated primarily from studies with small sample sizes and/or unadjusted comparisons between CKD patients and those without CKD. In addition, most of them considered elderly subjects and/or CKD patients, where significantly increased levels of serum adiponectin were found [15,16,17,18]. Furthermore, some studies have proposed that elevated serum adiponectin levels may be considered a prognostic marker in the progression of CKD due to the negative correlation with glomerular filtration [17]. In addition, adiponectin is significantly positively associated with severity of CKD measured by eGFR and urinary albumin [33]. However, to our knowledge, there are no studies that have considered the regulation of adiponectin levels in ADPKD patients.

Here, we found that, contrary to what is reported in CKD, adiponectin is statistically lower in ADPKD patients compared to healthy controls. Importantly, adiponectin levels are directly correlated with EGFR in our cohort of ADPKD patients and are further lower in ESRD patients compared to non-ESRD patients, indicating a relationship between adiponectin and renal health and an inverse relation with ADPKD severity. The literature data that report adiponectin up-regulation in renal diseases, but no data are available for a difference in ADPKD. Functionally, adiponectin role in kidney disorders have been related to multiple factors, i.e., the regulation of inflammatory processes in nephrons, involvement in vascular calcifications, and in the control of the nutritional status [34]. This study fails to clarify the functional role of adiponectin in ADPKD, but the data obtained, such as the different modulation of adiponectin in ESRD patients, and the direct correlation with eGFR, together with the inverse relationship with creatinine and albumin, suggest that the expression of this adipokine may be related to renal functioning. It is noticeable that, to our knowledge, there is no evidence of a correlation between adiponectin and serum albumin, but serum adiponectin levels are reported elevated in patients with albuminuria and positively correlated with the urine albumin/creatinine ratio [18].

On the other hand, in type I diabetic and diabetic nephropathy patients, it has been demonstrated that adiponectin predicts all-cause mortality and ESRD [17].

From this perspective, adiponectin may represent a central messenger in establishing a cross-talk between adipose tissue and kidney; furthermore, based on our data and those present in the literature, it could be hypothesized that adiponectin acts as an anti-inflammatory cytokine and, therefore, participates in the progression of the disease.

Several genetic polymorphisms have been described to have a role in the regulation of adiponectin levels [35]; consequently, we analyzed the most relevant SNPs in ADIPOQ and PPARγ genes, finding 14 patients bearing the PPARγ rs1801282 SNP and 29 patients bearing the ADIPOQ SNP (rs1501299). The PPAR SNP rs1801282, as reported in the study by Zusi et al., has a distribution in the Italian population of 12% in heterozygosity, comparable to that found in the present paper (15%) in ADPKD Italian patients [36]. Interestingly, PPARγ mutated patients had higher adiponectin levels compared to negative patients; such data is in accordance with previous studies that described higher levels of adiponectin in relation to the PPARγ SNP in several diseases, such as diabetes [37,38]. However, it is important to notice that, for the first time, we described this association in a renal disorder. Adiponectin expression is clearly related to PPARγ, since administration of the synthetic PPARγ ligands, the thiazolidinediones (TZDs), increases adiponectin concentrations [39]. Interestingly, the PPARγ agonist pioglitazone is used such as a drug for PKD patients and, interestingly, its insulin-sensitizing effects are partly mediated by the increased expression of adiponectin [40]. Considering ADIPOQ mutated patients, Pileggi et al. described a frequency of 12% and 41% of homozygous and heterozygous patients, respectively, in Italian subjects [41]; here, we report a 5.4% of frequency for homozygous and 26% for heterozygous patients. The rs1501299 SNP was previously analyzed in several populations, all describing the mutated genotype as associated with lower adiponectin levels [42]. Indeed, after a hypocaloric diet, the mutated genotype of an ADIPOQ gene variant (rs1501299) was related to a worse improvement in adiponectin levels within a cohort of obese patients [43]. Similar results were reported in healthy school-aged children of Greek origin under a fiber diet [44]. In addition, the distribution of the ADIPOQ rs1501299 significantly differs between CKD and controls. However, although several studies considered metabolic diseases, to our knowledge, this is the first study considering a population characterized by renal disease. Recently, a family harboring a 10-nucleotide deletion mutation in ADIPOQ was reported to co-segregate with diabetes and end-stage renal disease. This mutation introduces a frameshift in exon 3, resulting in a premature termination codon that disrupts translation of adiponectin’s globular domain [45].

Adiponectin exerts its effects via interaction with two specific receptors, AdipoR1 and AdipoR2 [46]. Previously, a significant association between ADIPOR2 SNPs and eGFR have been identified suggesting a potential effect of ADIPORs gene variants on kidney function [46]. Therefore, here we investigated the principal SNPs in ADIPOR1-2 genes in our ADPKD population in relation to biochemical and clinical parameters, but no relevant associations were found with biochemical parameters. Kobayashi et al. described several AdipoR2 SNPs associated with kidney function, suggesting that the effects of this polymorphism on adiponectin receptor may affect kidney function in the elderly Japanese population [47]. However, in our population, these SNPs were not present. Overall, our results indicated that determining genetic background could benefit ADPKD patient management and expand the number of genetic susceptibility biomarkers.

It is important to consider that our study does have several limitations: first, our study missed follow-up information; secondly, genetic background is missing in the control subjects, and this might weaken the discriminatory statistical power to identify true associations of genetic variants and clinical characteristics. 

## 5. Conclusions

In conclusion, this report demonstrated that serum adiponectin levels could influence the clinical phenotype of ADPKD patients, and that to analyze the association between polymorphisms and ADPKD risk could potentially provide important insights into ameliorating kidney outcome. Our results suggest that additional longitudinal studies and clinical trials should be conducted to investigate if adipocytokines play a role in the development and progression of ADPKD. Further studies that focus on the adiponectin pathway and gene–environment interactions ADPKD patients will aid us in obtaining a deeper understanding of adiponectin role in the establishment and prognosis of PKDs. Studies with larger sample sizes are required to improve the precision of point estimates.

## Figures and Tables

**Table 1:** Comparison of the biochemical and clinical features of the study participants.

Parameter		ADPKD *n* = 92	Controls *n* = 104	*p*-Value
		Mean (SD)	Mean (SD)	
Age (years)		42.84 (14.7)	45.96 (16.6)	0.169
Sex	F	49	48	0.86
	M	43	56	
BMI (kg/m^2^)		25.81 (4.8)	24.66 (2.5)	0.065
Systolic pressure (mmHg)		132.89 (16.7)	123.66 (3.35)	**0.006**
Diastolic pressure (mmHg)		82.35 (10.3)	83.66 (5.23)	0.50
Total cholesterol (mg/dL)		178.48 (35.9)	185.84 (33.1)	0.06
Triglycerides (mg/dL)		101.34 (67.3)	94.06 (50.51)	0.39
Glycemia (mg/dL)		81.47 (17.3)	83.96 (15.0)	0.29
AST (U/L)		20.66 (6.4)	20.36 (6.5)	0.74
ALT (U/L)		20.04 (11.4)	22.15 (15.9)	0.08
WBC (×10^3^/μL)		6.91 (2.0)	6.57 (1.5)	0.30
Neutrophils %		60.20 (10.2)	56.91 (9.6)	**0.02**
Lymphocytes %,		28.15 (7.8)	32.63 (8.2)	**0.00**
NLR		2.46 (1.3)	1.95 (0.9)	**0.004**
RBC (×10^6^/μL)		4.66 (0.7)	4.92 (0.9)	0.06
HGB (g/dL)		15.27 (1.3)	14.09 (1.3)	0.40
Albumin (mg/dL)		4.55 (0.3)	4.70 (0.3)	**0.01**
Creatinine (mg/dL)		1.29 (1.1)	0.91 (0.2)	**0.001**
Uric Acid (mg/dL)		5.45 (1.6)	4.79 (1.2)	**0.005**
Urea (mg/dL)		49.89 (30.2)	36.20 (9.5)	**0.000**
EGFR (mL/min/1.73 m^2^)		78.99 (33.3)	90 (6.7)	

**Table 2:** Adiponectin comparison between ADPKD patients and controls after adjustment for age, body weight, BMI, systolic pressure, and diastolic pressure.

Adjusted		Adiponectin						*p*
		Controls			ADPKD			
		Mean		ES	Mean	±	ES	
Age	years	16.7	±	0.402	14.1	±	0.425	0.00001
Body Weight	kg	16.6	±	0.414	14.3	±	0.431	0.0003
BMI	kg/m^2^	16.7	±	0.404	14.2	±	0.428	0.00004
Systolic pressure	mmHG	17.0	±	0.717	14.3	±	0.445	0.002
Diastolic Pressure	mmHG	17.1	±	0.700	14.3	±	0.441	0.0009

**Table 3:** PPARγ, ADIPOQ, ADIPOR1, ADIPOR2 gene polymorphisms distribution in ADPKD population.

Gene	SNP (rs Number)	*n* Patients	Het/Homo	Age	Adiponectin (µg/mL) (st.dev)	eGFR (mL/min/1.73 mq) (st.dev)	Systolic Blood Pressure (mm/Hg) (st.dev)	Diastolic Blood Pressure (mm/Hg) (st.dev)
PPARγ	c.34 C>G (rs1801282)	14	14/0	46 (15.35)	15.92 (3.27)	65.21 (36.87)	133.30 (22.65)	83.52 (18.18)
ADIPOQ	c.45C>T (rs2241766)	26	20/6	43 (14.84)	14.11 (4.39)	66.18 (31.85)	136.09 18.17)	82.97 (9.49)
	c.268G>A (rs62625753)	4	4/0	58 (18.6)	14.25 (5.00)	50.25 (37.73)	136.67 (5.77)	88.33 (7.63)
	c.214+62G>T (rs1501299)	29	24/5	43.7(16.28)	14.5 (4.42)	78.3 (35.15)	134.0 (18.11)	84.4 (10.21)
ADIPOR1	c. 94-8T>G (rs2275737)	23	17/6	44 (12.92)	13.25 (3.86)	64.34 (31.36)	134.14 (13.36)	82.14 (8.16)
	c. 94-12>G (rs2275738)	25	18/7	44 (12.92)	13.25 (3.86)	64.34 (31.36)	134.14 (13.36)	82.14 (8.16)
ADIPOR2	c.795G>A (rs16928751)	18	16/2	44 (13.60)	14.11 (3.97)	67.80 (38.35)	133.53 (13.89)	84.12 (10.64)
	c.1718C>T (rs1044471)	23	17/6	41 (12.65)	12.98 (3.56)	68.99 (33.83)	132.32 (13.50)	80.89 (8.39)
	c.1642C>T (rs12342)	16	13/3	45 (14.47)	14.57 (4.39)	68.86 (34.50)	134.25 (15.83)	82.97 (9.53)

## Data Availability

Data are contained within the article.

## Supplementary Materials

The following supporting information can be downloaded at: https://www.mdpi.com/article/10.3390/genes15040484/s1, Table S1: Genetic characterization of ADPKD patients analyzed in the study. PKD1 and PKD2 gene mutations were shown. Table S2: SNPs in PPARg, ADIPOQ, ADIPOR1, ADIPOR2 genes.

## References

[1] Satariano M., Ghose S., Raina R. (2024). The Pathophysiology of Inherited Renal Cystic Diseases. Genes.

[2] Harris P.C., Torres V.E. (2009). Polycystic kidney disease. Annu. Rev. Med..

[3] Ekinci I., Buyukkaba M., Cinar A., Tunc M., Cebeci E., Gursu M., Kazancioglu R. (2021). Endothelial Dysfunction and Atherosclerosis in Patients With Autosomal Dominant Polycystic Kidney Disease. Cureus.

[4] Azurmendi P.J., Fraga A.R., Galan F.M., Kotliar C., Arrizurieta E.E., Valdez M.G., Forcada P.J., Stefan J.S., Martin R.S. (2009). Early renal and vascular changes in ADPKD patients with low-grade albumin excretion and normal renal function. Nephrol. Dial. Transplant..

[5] Pandita S., Khullar D., Saxena R., Verma I.C. (2018). Autosomal Dominant Polycystic Kidney Disease: Presence of Hypomorphic Alleles in PKD1 Gene. Indian J. Nephrol..

[6] Riccio E., Imbriaco M., Daniele A., Iaccarino G., Pisani A. (2023). The Case|A patient with autosomal dominant polycystic kidney disease with an atypical kidney magnetic resonance image. Kidney Int..

[7] Prasad R., Jha R.K., Keerti A. (2022). Chronic Kidney Disease: Its Relationship With Obesity. Cureu.

[8] Serrano E., Shenoy P., Cantarin M.P. (2023). Adipose tissue metabolic changes in chronic kidney disease. Immunometabolism.

[9] Nigro E., Scudiero O., Monaco M.L., Palmieri A., Mazzarella G., Costagliola C., Bianco A., Daniele A. (2014). New insight into adiponectin role in obesity and obesity-related diseases. BioMed Res. Int..

[10] Sun Y., Xun K., Wang C., Zhao H., Bi H., Chen X., Wang Y. (2009). Adiponectin, an unlocking adipocytokine. Cardiovasc. Ther..

[11] Wang Z.V., Scherer P.E. (2016). Adiponectin, the past two decades. J. Mol. Cell Biol..

[12] Corbi G., Polito R., Monaco M.L., Cacciatore F., Scioli M., Ferrara N., Daniele A., Nigro E. (2019). Adiponectin Expression and Genotypes in Italian People with Severe Obesity Undergone a Hypocaloric Diet and Physical Exercise Program. Nutrients.

[13] Hassun L.A., Ruggeri M.L.R., de Souza S.A., Rossato A.M., Chmieleski G.S., de Carvalho L.S., Riccetto A.G.L., Degasperi G.R. (2023). Adipokines from adipose tissue and common variable immunodeficiency: Is there any association?. Scand. J. Immunol..

[14] Lin Y.-H., Jiang T.-X., Hu S.-X., Shi Y.-H. (2020). Association between serum adiponectin concentrations and chronic obstructive pulmonary disease: A meta-analysis. Biosci. Rep..

[15] Heidari M., Nasri P., Nasri H. (2015). Adiponectin and chronic kidney disease; a review on recent findings. J. Nephropharmacol..

[16] Vahdat S. (2018). The complex effects of adipokines in the patients with kidney disease. J. Res. Med. Sci..

[17] Przybyciński J., Dziedziejko V., Puchałowicz K., Domański L., Pawlik A. (2020). Adiponectin in Chronic Kidney Disease. Int. J. Mol. Sci..

[18] Song S.H., Oh T.R., Choi H.S., Kim C.S., Ma S.K., Oh K.H., Ahn C., Wan Kim S., Bae E.H. (2020). High serum adiponectin as a biomarker of renal dysfunction: Results from the KNOW-CKD study. Sci. Rep..

[19] Shoji T., Shinohara K., Hatsuda S., Kimoto E., Fukumoto S., Emoto M., Tahara H., Koyama H., Ishimura E., Miki T. (2005). Altered relationship between body fat and plasma adiponectin in end-stage renal disease. Metabolism.

[20] Menon V., Li L., Wang X., Greene T., Balakrishnan V., Madero M., Pereira A.A., Beck G.J., Kusek J.W., Collins A.J. (2006). Adiponectin and mortality in patients with chronic kidney disease. J. Am. Soc. Nephrol..

[21] Siitonen N., Pulkkinen L., Lindström J., Kolehmainen M., Eriksson J.G., Venojärvi M., Ilanne-Parikka P., Keinänen-Kiukaanniemi S., Tuomilehto J., Uusitupa M. (2011). Association of ADIPOQ gene variants with body weight, type 2 diabetes and serum adiponectin concentrations: The Finnish Diabetes Prevention Study. BMC Med. Genet..

[22] Hivert M.-F., Manning A.K., McAteer J.B., Florez J.C., Dupuis J., Fox C.S., O’Donnell C.J., Cupples L.A., Meig J.B. (2008). Common variants in the adiponectin gene (ADIPOQ) associated with plasma adiponectin levels, type 2 diabetes, and diabetes-related quantitative traits: The Framingham Offspring Study. Diabetes.

[23] Christodoulou A., Ierodiakonou D., Awofala A.A., Petrou M., Kales S.N., Christiani D.C., Mantzoros C.S., Christophi C.A. (2020). Variants in ADIPOQ gene are linked to adiponectin levels and lung function in young males independent of obesity. PLoS ONE.

[24] Yuan Y., Jiang H., Kuang J., Hou X., Feng Y., Su Z. (2012). Genetic variations in ADIPOQ gene are associated with chronic obstructive pulmonary disease. PLoS ONE.

[25] Chen H.-H., Huang Y.-L., Chen M.-C., Wu C.-Y., Lin Y.-C., Shiue H.-S., Hsu S.-L., Hsueh Y.-M. (2023). Chronic Kidney Disease: Interaction of Adiponectin Gene Polymorphisms and Diabetes. Int. J. Mol. Sci..

[26] Zha D., Wu X., Gao P. (2017). Adiponectin and Its Receptors in Diabetic Kidney Disease: Molecular Mechanisms and Clinical Potential. Endocrinology.

[27] Guo M., Li C., Lei Y., Xu S., Zhao D., Lu X.-Y. (2017). Role of the adipose PPARŒ≥-adiponectin axis in susceptibility to stress. Mol. Psychiatry.

[28] Nigro E., Amicone M., D’arco D., Sellitti G., De Marco O., Guarino M., Riccio E., Pisani A., Daniele A. (2023). Molecular Diagnosis and Identification of Novel Pathogenic Variants in a Large Cohort of Italian Patients Affected by Polycystic Kidney Diseases. Genes.

[29] Becic T., Studenik C., Hoffmann G. (2018). Exercise Increases Adiponectin and Reduces Leptin Levels in Prediabetic and Diabetic Individuals: Systematic Review and Meta-Analysis of Randomized Controlled Trials. Med. Sci..

[30] Signoriello E., Lus G., Polito R., Casertano S., Scudiero O., Coletta M., Monaco M.L., Rossi F., Nigro E., Daniele A. (2019). Adiponectin profile at baseline is correlated to progression and severity of multiple sclerosis. Eur. J. Neurol..

[31] Christou G.A., Kiortsis D.N. (2014). The role of adiponectin in renal physiology and development of albuminuria. J. Endocrinol..

[32] Kim H.Y., Bae E.H., Ma S.K., Chae D.W., Choi K.H., Kim Y.-S., Hwang Y.-H., Ahn C., Kim S.W. (2016). Association of serum adiponectin level with albuminuria in chronic kidney disease patients. Clin. Exp. Nephrol..

[33] Sweiss N., Sharma K. (2014). Adiponectin effects on the kidney. Best Pract. Res. Clin. Endocrinol. Metab..

[34] Jia T., Carrero J.J., Lindholm B., Stenvinkel P. (2012). The complex role of adiponectin in chronic kidney disease. Biochimie.

[35] Howlader M., Sultana I., Akter F., Murad Hossain M. (2021). Adiponectin gene polymorphisms associated with diabetes mellitus: A descriptive review. Heliyon.

[36] Zusi C., Rioda M., Maguolo A., Emiliani F., Unali I., Costantini S., Corradi M., Contreas G., Morandi A., Maffeis C. (2023). IGF1 and PPARG polymorphisms are associated with reduced estimated glomerular filtration rate in a cohort of children and adolescents with type 1 diabetes. Acta Diabetol..

[37] von Frankenberg A.D., Reis A., Gerchman F. (2017). Relationships between adiponectin levels, the metabolic syndrome, and type 2 diabetes: A literature review. Arch. Endocrinol. Metab..

[38] AlSaleh A., Sanders T.A.B., O’Dell S.D. (2012). Effect of interaction between PPARG, PPARA and ADIPOQ gene variants and dietary fatty acids on plasma lipid profile and adiponectin concentration in a large intervention study. Proc. Nutr. Soc..

[39] Riera-Guardia N., Rothenbacher D. (2008). The effect of thiazolidinediones on adiponectin serum level: A meta-analysis. Diabetes Obes. Metab..

[40] Mao Z., Valluru M.K., Ong A.C.M. (2021). Drug repurposing in autosomal dominant polycystic kidney disease: Back to the future with pioglitazone. Clin. Kidney J..

[41] Pileggi S., Barlera S., Nicolis E., Crociati L., Pietri S., Specchia C., Franzosi M.G. (2014). Association of ADIPOQ variants and heart failure in an Italian population. Ther. Adv. Cardiovasc. Dis..

[42] de Luis D.A., Izaola O., de la Fuente B., Primo D., Ovalle H.F., Romero E. (2016). rs1501299 Polymorphism in the Adiponectin Gene and Their Association with Total Adiponectin Levels, Insulin Resistance and Metabolic Syndrome in Obese Subjects. Ann. Nutr. Metab..

[43] Aller R., Izaola O., Primo D., de Luis D.A. (2019). The effect of single-nucleotide polymorphisms at the ADIPOQ gene locus rs1501299 on metabolic parameters after 9 mo of a high-protein/low-carbohydrate versus a standard hypocaloric diet. Nutrition.

[44] Ntalla I., Dedoussis G., Yannakoulia M., Smart M.C., Louizou E., Sakka S.D., Papoutsakis C., Talmud P.J. (2009). ADIPOQ gene polymorphism rs1501299 interacts with fibre intake to affect adiponectin concentration in children: The GENe-Diet Attica Investigation on childhood obesity. Eur. J. Nutr..

[45] Simeone C.A., Wilkerson J.L., Poss A.M., Banks J.A., Varre J.V., Guevara J.L., Hernandez E.J., Gorsi B., Atkinson D.L., Turapov T. (2022). A dominant negative ADIPOQ mutation in a diabetic family with renal disease, hypoadiponectinemia, and hyperceramidemia. NPJ Genom. Med..

[46] Kim Y., Park C.W. (2019). Mechanisms of Adiponectin Action: Implication of Adiponectin Receptor Agonism in Diabetic Kidney Disease. Int. J. Mol. Sci..

[47] Kobayashi H., Otsuka H., Yanai M., Hara M., Hishiki M., Soma M., Abe M. (2019). Adiponectin Receptor gene Polymorphisms are Associated with Kidney Function in Elderly Japanese Populations. J. Atheroscler. Thromb..

