# Supplementary material for: Exploring Adiponectin in Autosomal Dominant Kidney Disease: Insight and Implications
Source: Genes (Basel). 2024 Apr 11;15(4):484. doi: 10.3390/genes15040484 (PMC11050174; doi:10.3390/genes15040484)
Supplement: Supplementary file 1 [file genes-15-00484-s001.zip › genes-2926202-supplementary.pdf]

Gene	cDNA change	Region	Protein variant	ACMG Classification
PKD1	c.359+2T>G	Int3		P
	c.427C>T	Ex4	p.Gln143Ter	LP
	c.680_681insT (?)	Ex5	p.Gln227Hisfs*34	LP
	c.1105_1106delAG	Ex5	p.Ser369Ter	P
	C.1141G>A	Ex5	p.Gly381Ser	P
	c.1273_1275delGAG	Ex6	p.Glu425del	LP
	c.10722G>A	Ex7	p.Trp3574Ter	P
	c.1832G>C	Ex9	p.Arg611Pro	Vus
	c.2085delC	Ex10	p.Ala696ArgfsTer89	P
	c.2180T>C	Ex11	p.Leu727Pro	LP
	c.2189C>T	Ex11	p.Ser730Leu	VUS
	c.2215dupC	Ex11	p.Gln739ProfsTer59	LP
	c.2534T>C	Ex11	p.Leu845Ser	P
	c.2711_2712delAG	Ex11	p.Glu904GlyfsTer196	LP
	c.2985+3A>C	Int12		VUS
	c.3067C>T	Ex13	p.Gln1023Ter	P
	c.3295+2T>C	Int14	-	P
	c.6916-9G>A	Int15	-	LP
	c.3349C>T	Ec15	p.Gln1117Ter	P
	c.3398_3399delTG	Ex15	p.Val1133GlufsTer2	LP
	c.3514C>T	Ex15	p.Gln1172Ter	P
	c.3520_3527delCAGCCGGC	Ex15	p.Gln1174Cysfs34Ter	LP
	c.3706C>T	Ex15	p.Gln1236Ter	P
	c.3745delG	Ex15	p.Asp1249ThrfsTer24	P
	c.3802C>T	Ex15	p.Gln1268Ter	LP
	c.3955G>A	Ex15	p.Gly1319Arg	P
	c.4888C>T	Ex15	p.Gln1630*	P
	c.4951C>T	Ex15	p.Gln1651Ter	P
	c.5154_5163dupGGGGTGGCTG	Ex15	p.TerMet1722GlyfsTer52	P
	c.5223dupG	Ex15	p.Leu1742AlafsTer29	LP
	c.5609A>G	Ex15	p.Asn1870Ser	LP
	c.5869_5870dupAG	Ex15	p.Ser1957ArgfsTer16	LP
	c.5884C>T	Ex15	p.Gln1962Ter	P
	c.5905G>T	Ex15	p.Glu1969Ter	P
	c.5911G>A	Ex15	p.Val1971Met	VUS
	c.6199C>T	Ec15	p.Gln2067Ter	P
	c.7416_7417insC	EX18	p.Gly2473ArgfsTer28	LP
	c.7597_7598delTC	Ex19	p.Ser2533Glnfs*61	P
	c.7864_7899del	Int20-Ex21	p.Tyr2622_Lys2633del	p
	c.7984C>T	Ex21	p.Gln2662Ter	LP
	c.8238delG	Ex23	p.Met2747TrpfsTer9	LP
	c.8279T>G	Ex23	p.Met2760Arg	LP
	c.8311G>A	Ex23	p.Glu27771 Lys	P

	c.8371_8372dupCG	Ex23	p.Ser2792GlyfsTer84	P
	c.8698C>T	Ex23	p.Gln2900Ter	P
	c.8935_8937delTTC	Ex24	p.Phe2979del	P
	c.9404C>T	ex27	p.Thr3135Met	VUS
	c.9425_9426insA	Ex27	p.Tyr3143ValfsTer36	P
	c.9562A>G	Ex27	p.Asn3188Asp	VUS/LP
	c.9564_9566delCAA	Ex27	p.Asn3188del	LP
	c.9676A>G	Ex28	p.Asn3226Asp	V
	c.9771_9774delCTTT	Ex29	p.Phe3257LeufsTer58	P
	c.10026delT	Ex30	p.Leu3343SerfsTer54	P
	c.10217+2T>G	Int32	-	P
	c.10420C>T	Ex34	p.Gln3474Ter	P
	c.10459C>T	Ex34	p.Gln3487Ter	P
	c.10549G>T	Ex35	p.Glu3517Ter	LP
	c.10591C>T	Ex35	p.Gln3531Ter	P
	C.10722G>A	EX36	p.Trp3574Ter	P
	c.10768T>C	Ex36	p.Ser3590Pro	V
	c.10807G>C	Ex36	p.Glu3603Gln	LP
	c.10894_10895del	Ex37	p.Ser3632ProfsTer88	P
	c.10973_10987delAAGAA GCCCGCAAGG	Ex37	p.Glu3658_Lys3662del	LP
	c.11267-1G>T	Int39	-	p
	c.11438_11439delAT	Ex41	p.Tyr3813Ter	LP
	c.11534G>T	Ex41	p.Arg3845Met	P
	c.11571C>G	Ex42	p.Tyr3857Ter	P
	c.11585T>G	Ex42	p.Leu3862Arg	VUS
	c.11639_11683del	Ex42	p.Ala3880_Ala3894del	VUS
	c.11646_11659del	Ex42	p.Ser3883CysfsTer72	P
	c.11705_11708delCCTC	Ex42	p.Thr3902ArgfsTer41	LP
	c.11967_11974dup	Ex43	p.Ser3992TrpfsTer49	P
	c.11881C>T	Ex43	p.Gln3961Ter	P
	c.12008dupA	Ex 44	p.Gln4005AlafsTer152	P
	c.12058C>T	Ex44	p.Arg4021Ter	P
	c.12908A>T	Ex46	p.4303LeuextTer35	VUS
	c.8267C>T	Ex23	p.Thr2756Ile	VUS
	c.11870G>A	Ex43	p.Gly3957Asp	VUS
PKD2	c.595+3A>T	Int1	-	P
	c.261G>A	Ex1	p.Trp87Ter	P
	c.709+1G>A	Int2	-	P
	c.608C>T	Ex2	p.Thr203Ile	VUS
	c.637C>T	Ex2	p.Arg213Ter	p
	c.1094+3_1094+6delAAGT	Int4	-	P
	c.916C>T	Ex4	p.Arg306Ter	p
	c.958C>T	Ex4	p.Arg320Ter	P
	c.964C>T	Ex4	p.Arg322Trp	P
	c.1142G>T	Ex5	p.Gly381Val	LP

c.1244T>G	Ex5	p.Leu415Arg	LP
c.1395T>A	Ex6	p.Tyr465Ter	LP
c.1837C>T	Ex8	p.Gln613Ter	P
c.2117delA	Ex10	p.Lys706ArgfsTer10	LP
c.2358delG	Ex12	p.Glu787ArgfsTer14	P
c.2419C>T	ex13	p.Arg807Ter	P
c.2670+5G>A	Int 14	-	VUS
c.2614C>T	Ex14	p.Arg872Ter	P

Supplementary Table S1: Genetic characterization of ADPKD patients analyzed in the study. PKD1 and PKD2 gene mutations were shown.

Gene	SNPs	cDNA	Protein	Location	Coding impact	Publications
PPARγ NM_001354668.2	rs1801282	c.34 C>G	P12A p.(Pro12Ala)	exon 1 of 7 position 210 of 258 (coding)	Missense	DOI: 10.1515/CCLM.2009.242. DOI: 10.1038/ng.3943.
ADIPOQ NM_001177800.1	rs2241766	c.45T>G	G15= p.(Gly15=)	exon 3 of 4 position 53 of 222 (coding)	synonymous	DOI:10.1016/j.jdiacomp.2012.02.008 DOI: 10.1371/journal.pone.0058412.
	rs1501299	c.214+62G>T	-	intron 3 of 3 position 62 of 911 (intronic)	-	DOI: 10.1186/1471-2350-13-40. DOI: 10.1016/j.mce.2011.10.001.
	rs62625753	c.268G>A	G90S p.(Gly90Ser)	exon 4 of 4 position 54 of 4280 (coding)	missense	DOI: 10.3390/nu11092195. DOI: 10.1016/j.cca.2008.02.011.
	rs1295862787	c.696C>T	S232= p.(Ser232=)	exon 4 of 4 position 482 of 4280 (coding)	synonymous	-
ADIPOR1 NM_001290557.1	rs2275737	c. 94-8T>G	-	intron 1 of 7 position 7013 of 7020 (splicing, intronic)	-	DOI: 10.1900/RDS.2017.14.311. PMID: 26629210
	rs2275738	c. 94-12A>G	-	intron 1 of 7 position 7009 of 7020 (intronic)	-	DOI: 10.1016/j.mgene.2016.07.008
ADIPOR2 NM_024551.2	rs16928751	c.795G>A	synonymous	exon 6 of 8 position 145 of 188 (coding)	Q265= p.(Gln265=)	DOI: 10.1900/RDS.2008.5.28 DOI: 10.1186/1475-2840-10-83
	rs1044471	c.*1718C>T o g.33447T	-	exon 8 of 8 (3'UTR) position 1847 of 2731	-	DOI: 10.1016/j.gene.2018.03.022 DOI: 10.1007/s12032-013-0658-9 DOI: 10.1038/oby.2008.344
	rs12342	c.*1642C>T	-	exon 8 of 8 (3'UTR) position 1771 of 2731	-	DOI: 10.1016/j.ygeno.2019.12.020 DOI: 10.4238/2014.September.26.19
	rs767870	c.650+20G>A	-	intron 5 of 7 position 20 of 251 (intronic)	-	DOI: 10.2337/diabetes.55.03.06.db05-0665 DOI: 10.1530/EJE-08-0900

Supplementary Table S2: SNPs in PPARγ, ADIPOQ, ADIPOR1, ADIPOR2 genes.
